# DNI-MDCAP: improvement of causal MiRNA-disease association prediction based on deep network imputation

**DOI:** 10.1186/s12859-024-05644-6

**Published:** 2024-01-12

**Authors:** Yu Han, Qiong Zhou, Leibo Liu, Jianwei Li, Yuan Zhou

**Affiliations:** 1 Department of Biomedical Informatics, School of Basic Medical Sciences, Peking University, Beijing, China; 2 Institute of Computational Medicine, School of Artificial Intelligence, Hebei University of Technology, Tianjin, China; 3 State Key Laboratory of Vascular Homeostasis and Remodeling, Peking University, Beijing, China

**Keywords:** miRNA, miRNA-disease association, Causal miRNA-disease association prediction, Network imputation, Deep graph embedding

## Abstract

**Background:**

MiRNAs are involved in the occurrence and development of many diseases. Extensive literature studies have demonstrated that miRNA-disease associations are stratified and encompass ~ 20% causal associations. Computational models that predict causal miRNA-disease associations provide effective guidance in identifying novel interpretations of disease mechanisms and potential therapeutic targets. Although several predictive models for miRNA-disease associations exist, it is still challenging to discriminate causal miRNA-disease associations from non-causal ones. Hence, there is a pressing need to develop an efficient prediction model for causal miRNA-disease association prediction.

**Results:**

We developed DNI-MDCAP, an improved computational model that incorporated additional miRNA similarity metrics, deep graph embedding learning-based network imputation and semi-supervised learning framework. Through extensive predictive performance evaluation, including tenfold cross-validation and independent test, DNI-MDCAP showed excellent performance in identifying causal miRNA-disease associations, achieving an area under the receiver operating characteristic curve (AUROC) of 0.896 and 0.889, respectively. Regarding the challenge of discriminating causal miRNA-disease associations from non-causal ones, DNI-MDCAP exhibited superior predictive performance compared to existing models MDCAP and LE-MDCAP, reaching an AUROC of 0.870. Wilcoxon test also indicated significantly higher prediction scores for causal associations than for non-causal ones. Finally, the potential causal miRNA-disease associations predicted by DNI-MDCAP, exemplified by diabetic nephropathies and hsa-miR-193a, have been validated by recently published literature, further supporting the reliability of the prediction model.

**Conclusions:**

DNI-MDCAP is a dedicated tool to specifically distinguish causal miRNA-disease associations with substantially improved accuracy. DNI-MDCAP is freely accessible at http://www.rnanut.net/DNIMDCAP/.

**Supplementary Information:**

The online version contains supplementary material available at 10.1186/s12859-024-05644-6.

## Background

MicroRNAs (MiRNAs) are small endogenous RNAs that play important gene-regulatory roles by interacting with the mRNAs of protein-coding genes to direct their post-transcriptional regulations [1, 2]. With the development of biological technologies, rapid accumulating research articles have demonstrated the involvement of miRNAs in various biological processes including but not limited to cell proliferation [3], metabolism [4], embryonic development [5], and diverse diseases like breast cancer [6], diabetic complications [7, 8] and heart failure [9]. Notably, the roles of miRNAs in diseases can be stratified into causal and non-causal ones, as they can either directly promote/inhibit disease progression [10], or serve as molecules that accompany the changes in disease status [11]. Causal miRNAs can be involved in pathogenic mechanisms and developments of multiple diseases such as Parkinson’s disease [12] and cardiovascular diseases [13, 14]. Also, a group of potentially pathogenic miRNAs have been identified in the human brain and central nervous system [15]. That is to say, the associations between miRNAs and diseases can be further categorized based on causality rather than simply considered binary. MiRNAs with causal associations to diseases can actively engage in diverse biological processes through targeting disease genes and other mechanisms. Once the regulation of a disease-causative miRNA is disrupted, the normal physiological regulation will be shifted to the pathological one, directly contributing to the onset and progression of the diseases [16]. Hence, causal miRNA-disease associations are of great prominence in investigating the molecular mechanisms of diseases and identifying miRNA candidates that can serve as novel targets for disease treatment.

Current experimental methods to determine miRNA-disease associations are often labor-intensive and time-consuming. Consequently, there is an urgent need to develop computational models that can more effectively predict the relation on a large scale. Exploiting various machine learning and graph-based methods, several previous studies have proposed computational models that can accurately identified general diseases-related miRNAs. For instance, Fu et al. [17] developed DeepMDA that extracted high-level features from similarity information using stacked autoencoders and then predicted miRNA-disease associations by adopting a 3-layer neural network. A sophisticated deep ensemble model proposed by Chen et al. [18] revealed potential miRNA-disease associations based on decision tree. Many other computational models, such as RWRMDA [19], NetCBI [20], similarly relied on complex graph algorithms to estimate the similarity links between miRNA and disease networks, thereby constructing models for miRNA-disease association prediction with the assumption that similar miRNAs tend to be associated with similar diseases. Liu et al. generated SMALF [21], learning latent miRNA and disease features through stacked autoencoders and utilizing XGBoost to predict miRNA-disease associations. Li et al. [22] advanced a new computational framework GATMDA to discover unknown miRNA-disease associations based on graph attention network with multi-source information, which effectively fuses linear and non-linear features. A novel method CFSAEMDA [23] captured the interactive features of miRNA and disease, applying stacked autoencoder and modified cascade forest model to complete the final prediction. It is not surprising that predicting disease-related miRNAs is an ongoing focus of research, but our previous benchmark study [24] has shown that most of the existing models were not fully capable of distinguishing causal miRNA-disease associations from non-causal associations. Causal inference has become an emerging and important topic in bioinformatics, like casual genetic association inference [25] and casual gene regulatory network inference [26]. To fill the gap of causal miRNA-disease association prediction, efforts to design dedicated computational models aimed at discerning causal miRNA-disease associations remain an urgent priority.

In the latest Human MicroRNA Disease Database (HMDD) v3.2 [27], Gao et al. [28] systematically annotated the miRNA-disease associations by manual reviewing of the literature, and finally 4294 causal miRNA-disease associations were labeled, which made it possible to train a computational model for causal miRNA-disease association prediction. Indeed, Gao et al. proposed the MiRNA-Disease Causal Association Predictor (MDCAP), which exploited class label propagation algorithm to predict potential miRNA disease causality. MDCAP showed a reliable predictive performance on distinguishing between miRNA-disease associations and unrelated miRNA-disease pairs (AUROC > 0.9). Nonetheless, the performance of MDCAP decreased significantly on the challenges in discriminating causal and non-causal miRNA-disease associations (AUROC = 0.695). To this ends, we have previously [29] introduced the Levenshtein-distance Enhanced MiRNA-Disease Causal Association Predictor (LE-MDCAP) that was built on Levenshtein distance estimation and matrix decomposition algorithm. Although this model demonstrated the ability to discriminate potential causal miRNAs-disease associations from non-causal ones (AUROC = 0.820), further improvements are still needed to improve the predictive performance. Wang et al. also developed DisiMiR [30], using network influence and miRNA conservation to predict causal miRNA for four specific diseases (breast cancer, Alzheimer’s disease, gastric cancer, and hepatocellular cancer). Nevertheless, additional efforts are necessary to establish a universally applicable predictive model for causal miRNA-disease associations.

In this study, we devised an improved prediction model for causal miRNA-disease association prediction, Deep Network Imputation-assisted MiRNA-Disease Causal Association Predictor (DNI-MDCAP), by the means of integrating multiple miRNA features and applying the deep graph embedding based-network imputation as well as graph semi-supervised learning algorithm. To demonstrate the effectiveness of our proposed approach, we conducted thorough evaluations through tenfold cross-validation and independent test, providing comprehensive measurements of our model’s performance. In addition, case studies were conducted to compare the prediction results with the latest experimental evidence that has not been recorded in the HMDD v3.2 database, further validating the reliability of our model.

## Implementation

### Overview of DNI-MDCAP workflow

To improve causal miRNA-disease association prediction, we adopted an integrated approach that combined various miRNA features and leveraged the network imputation method based on the deep graph embedding learning model, which finally led to the development of Deep Network Imputation-assisted MiRNA-Disease Causal Association Predictor (DNI-MDCAP) for predicting potential associations.

**Fig. 1:**
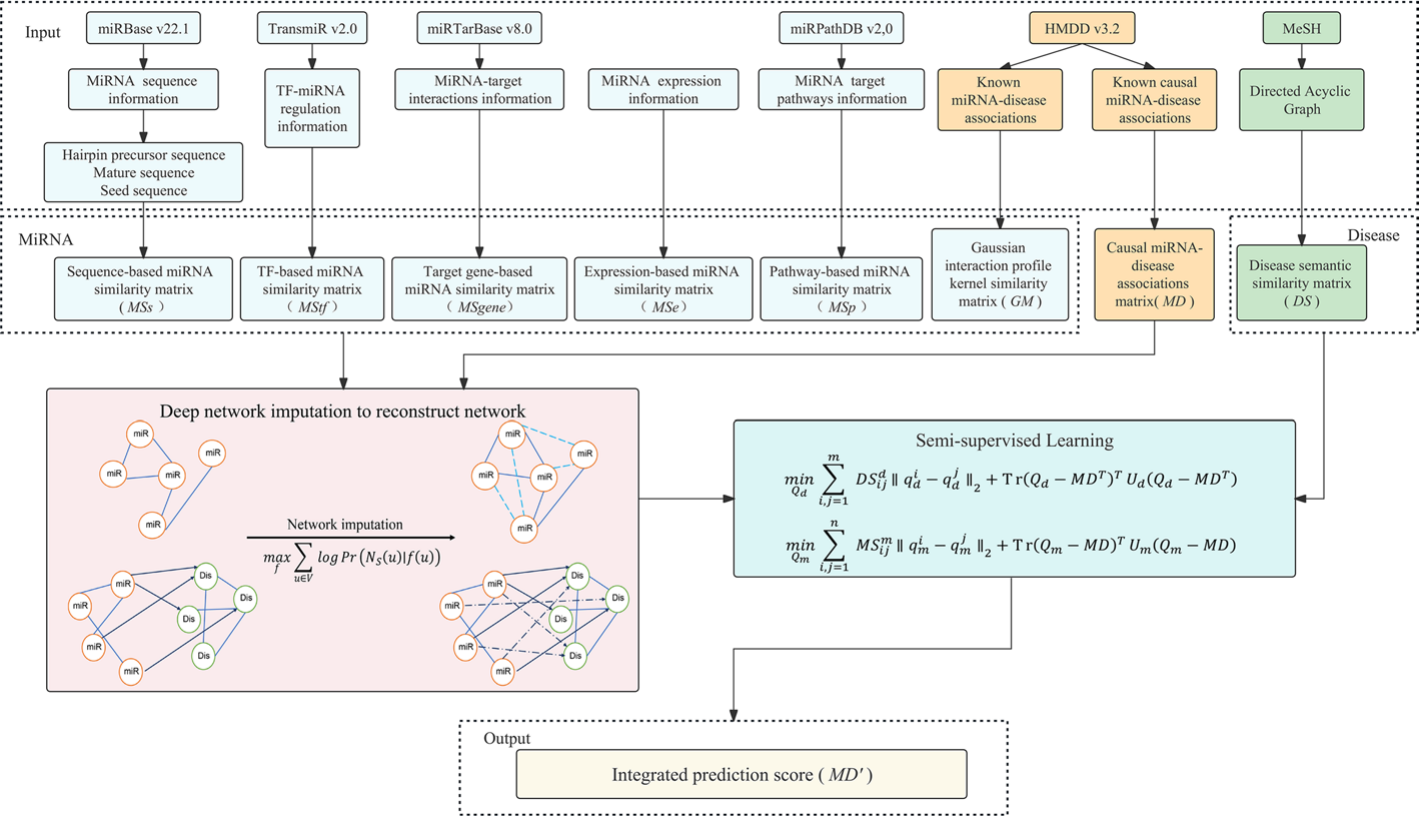
Workflow of DNI-MDCAP

The overall workflow of DNI-MDCAP is illustrated in Fig. 1. The workflow was consisted of several steps. Firstly, we obtained miRNA-related data from various databases, including sequence, transcription factor (TF), target gene, expression, pathway, and disease association information. According to the above miRNA-related data, we measured the similarity between any two miRNAs by Levenshitein distance, Tanimoto coefficient or Gaussian interaction profile kernel, and then integrated these miRNA similarity metrics into a final miRNA similarity matrix. The disease semantic similarity matrix and the known causal miRNA-disease association matrix were obtained from the hierarchical relationships between disease terms recorded in Medical Subject Headings (MeSH) database [31] and the known causality annotation from HMDD v3.2 [27, 28], respectively. Next, because current knowledge of causal miRNA-disease associations is still sparse, we employed the deep graph embedding learning-based link prediction algorithm to impute the missing links within the miRNA similarity and the causal miRNA-disease association networks. Lastly, we implemented the graph semi-supervised learning method to build models for each miRNA similarity matrix. The prediction scores from these models were then combined via a weighted sum approach to derive the final prediction results of DNI-MDCAP.

### Calculation of miRNA similarity

On accounting of our previous experience in establishing LE-MDCAP [29], Levenshtein distance was an informative method to calculate some of miRNA similarity metrics. Levenshtein distance is a measure of the degree of difference between two feature strings, also known as the edit distance. The similarity score $MS ({m}_{1}, {m}_{2})$ of miRNA ${m}_{1}$ and ${m}_{2}$ based on Levenshtein distance can be calculated as Eq. (1), where $LD{\prime}({m}_{1}, {m}_{2})$ indicates the minimum edit times to convert ${m}_{1}$ to ${m}_{2}$, and $len$ represents the length of the miRNA feature string.

1 $$\begin{array}{c}MS\left({m}_{1},{m}_{2}\right)=1-\frac{L{D}^{\mathrm{^{\prime}}}\left({m}_{1},{m}_{2}\right)}{{\text{len}}\left({m}_{1}\right)+{\text{len}}\left({m}_{2}\right)}\end{array}$$

The advantage of utilizing the Levenshtein distance as a measure of miRNA similarity, derived from Eq. (2), lies in its ability to effectively compare features in different scales.

2 $$\begin{array}{c}0\le L{D}^{\mathrm{^{\prime}}}\left({m}_{1},{m}_{2}\right)\le {\text{len}}\left({m}_{1}\right)+{\text{len}}\left({m}_{2}\right)\end{array}$$

Notably, $MS ({m}_{1}, {m}_{2}$) should be in the range of 0.5 to 1, as in this study we only considered the unidirectional editing distance from ${m}_{1}$ to ${m}_{2}$ in this study. The higher the score, the more similar the two miRNAs are.

For sequence-based miRNA similarity, we collected sequence information from the miRBase v22.1 [32] and employed the Levenshtein distance to measure the similarity between any two miRNAs at three levels: miRNA precursors, mature miRNAs and miRNA seed sequences. Accordingly, three sequence based-miRNA similarity metrics were obtained, namely ${MS}_{SP}$, ${MS}_{SM}$, and ${MS}_{SS}$. The resulting sequence-based miRNA similarity matrix was calculated on the weighted sum of the above three metrics, where the weight sum was 1 and optimized with a step size of 0.05, with the final selected combination weight ${MS}_{S}$ = 0.80 ${MS}_{SP}$+ 0.15 ${MS}_{SM}$+ 0.05 ${MS}_{SS}$.

In terms of TF-based and target gene-based miRNA similarity metrics, we fetched human TF-miRNA regulation data from TransmiR v2.0 [33] database and the experimentally validated microRNA-target interactions data from miRTarBase v8.0 [34] database. For each miRNA-TF/target pair, a score of 1 was used to indicate the existence of a known regulation between miRNA and a TF/target gene, and 0 otherwise. Again, Levenshtein distance was used to calculate the similarity between any pair of miRNAs represented by the binary strings of TF and target gene regulations, which are denoted as ${MS}_{tf}$ and ${MS}_{gene}$, respectively.

As for expression-based miRNA similarity, we obtained miRNA expression data for 137 cell types by organizing the research results of Lorenzi et.al [35], and normalized the expressions to eliminate the scaling differences. For pathway-based miRNA similarity, we downloaded the p-values of enrichment for miRNA target genes from the miRPathDB v2.0 [36] and retained pathways with at least three miRNAs with p-values of less than 0.05. Notably, the expression and pathway information are not binary but exhibits a continuous distribution. Therefore, we used the Tanimoto coefficient to calculate the similarity between miRNAs, denoted as ${MS}_{E}$ and ${MS}_{P}$. The similarity between miRNA ${m}_{i}$ and miRNA ${m}_{j}$ was calculated as Eq. (3):

3 $$\begin{array}{c}{S}_{ij}=\frac{{m}_{i}\cdot {m}_{j}}{{\Vert {m}_{i}\Vert }^{2}+{\Vert {m}_{j}\Vert }^{2}-{m}_{i}\cdot {m}_{j}}\end{array}$$

Unlike many other similarity measurement methods that rely solely on the magnitude of differences, the Tanimoto coefficient takes into account the distribution and overlap of values within the data, making it particularly suitable for capturing similarities in continuous data with different ranges and distributions. Moreover, we evaluated the influence of different miRNA similarity measurement methods on the predictive performance of the model and chose the similarity metrics mainly based on its performance in casual-versus-non-causal discrimination (Additional file 1: Table S1).

Finally, based on previous research [37, 38], we also constructed the commonly used Gaussian interaction profile kernel similarity matrix $GM$ for miRNAs as a baseline method for measuring miRNA similarity.

### Known causal miRNA-disease associations

The human causal miRNA-disease association dataset was downloaded directly from HMDD v3.2 database. To facilitate the comparison of performance between DNI-MDCAP and MDCAP, we used the same dataset, containing 4228 experimentally validated causal associations between 535 miRNAs and 302 diseases. We constructed a binary adjacency matrix $MD$ of $nm\times nd$ to better represent the causal relationship of miRNA and diseases, where $nm$ and $nd$ represent the number of miRNAs and diseases, respectively. Specifically, if miRNA $m\left(i\right)$ was confirmed to have a causal association with disease $d(i)$, the value of $MD(i,j)$ was 1, and 0 otherwise.

### Calculation of disease semantic similarity

We introduced the well-known Wang’s diseases semantic similarity [39] to construct a disease similarity matrix $DS$, with the help of the semantic topological relationships between diseases recorded in the MeSH database. In MeSH, the topology of disease can be described as a directed acyclic graph (DAG), in other words, ${DAG}_{D}= (D, {T}_{D}, {E}_{D})$, where ${T}_{D}$ represents the node including disease $D$ and its ancestral disease, ${E}_{D}$ represents the edges of all relationships of the ${DAG}_{D}$. The contribution of disease $d$ to the semantic value of disease $D$ can be represented by the Eq. (4), where $\Delta$ is the semantic contribution factor and is usually set to 0.5.

4 $$\left\{\begin{array}{cc}{D}_{D}(D)=1& \text{if }d=D\\ {D}_{D}(d)=max\left\{{\Delta }^{*}{D}_{D}\left({d}^{\mathrm{^{\prime}}}\right)\mid {d}^{\mathrm{^{\prime}}}\in \text{ children of }d\right\}& \text{if }d\ne D\end{array}\right.$$

The semantic value $DC\left(D\right)$ of the disease $D$ can be obtained by summing all the contributions of the ancestral disease and disease $D$, as Eq. (5).

5 $$\begin{array}{c}DC\left(D\right)=\sum_{d\in T\left(D\right)} {D}_{D}\left(d\right)\end{array}$$

Thus, the semantic similarity of diseases ${D}_{i}$ and ${D}_{j}$ was calculated as Eq. (6).

6 $$\begin{array}{c}{\text{DSS}}\left({D}_{i},{D}_{j}\right)=\frac{\sum_{d\in T\left({D}_{i}\right)\cap T\left({D}_{j}\right)} \left({D}_{{D}_{i}}\left(d\right)+{D}_{{D}_{j}}\left(d\right)\right)}{DC\left({D}_{i}\right)+DC\left({D}_{j}\right)}\end{array}$$

In the light of the above calculations, diseases that have more largely shared DAG structures will have higher semantic similarity scores.

### Deep network imputation

We applied the Node2Vec algorithm, which is based on deep graph embedding learning and random walk [40] to predict uncharted associations in the miRNA similarity network and miRNA-disease association network to enhance and complete the connections between nodes in these two types of networks. Particularly, we transformed each miRNA similarity matrix into a binary adjacency matrix by truncating at a certain threshold $\xi$. For each network, $\xi$ is optimized according to the best AUROC when distinguishing causal miRNA-disease associations in cross-validation. The thresholds for ${MS}_{S}$, ${MS}_{E}$, $GM$, ${MS}_{P}$, ${MS}_{gene}$, ${MS}_{tf}$ were optimized as 0.75, 0.91, 0.3, 0.60, 0.97, 0.95, respectively.

The objective function of deep network imputation is shown in Eq. (7), where $f$ is the mapping function of node $u$ mapped to an embedded vector, and $N (u)$ is the set of nearest neighbors of nodes $u$ sampled by the sampling strategy $S$.

7 $$\begin{array}{c}\underset{f}{max} \sum_{u\in V} {\text{logPr}}\left({N}_{S}\left(u\right)\mid f\left(u\right)\right)\end{array}$$

Optimization procedure for the specific objective function was described in detail in the article by Mikolov et al. [41], and is not repeatedly described here. Finally, the learned deep graph embedding features were used to predict new links, thus reconstructing the re-linked miRNA similarity networks and known causal miRNA-disease association network. To effectively showcase the advantages of network imputation, we compared the model performance of LE-MDCAP and DNI-MDCAP with and without network imputation (Additional file 1: Table S2). For both model, network imputation was helpful for performance improvement.

### Semi-supervised learning model

As mentioned above, we acquired the re-linked causal miRNA-disease association matrix $MD$ and six miRNA similarity matrices, namely ${MS}_{S}$, ${MS}_{E}$, $GM$, ${MS}_{P}$, ${MS}_{gene}$, ${MS}_{tf}$ by deep network imputation. By combining each miRNA similarity matrix *MS* with the causal miRNA-disease association matrix *MD* and the disease semantic similarity matrix $DS$, the semi-supervised learning method proposed by Liang et al. [42] can be used to separately predict the causal association scores of miRNAs and diseases.

Suppose $n$ and $m$ represent the number of miRNAs and diseases in the dataset, respectively. For the miRNA space, given the causal miRNA-disease association matrix $MD$ and the miRNA similarity matrix $MS$, an incidence matrix $Qm$ can be obtained to reflect the causal association probabilities between certain miRNAs and diseases. Then an objective function based on the norm and trace (Tr) was defined as Eq. (8), where ${q}_{m}^{i}$ and ${q}_{m}^{j}$ denote the *i*-th and *j*-th columns of ${Q}_{m}$. ${U}_{m}$ is a diagonal matrix in which the *i*-th diagonal element controls the effect of the initial association of $MD$. Intuitively, the objective here is to ensure that similar miRNAs have similar causal disease associations while optimizing the consistency between the predicted causal miRNA-disease associations with the known ones.

8 $$\begin{array}{c}\underset{{Q}_{m}}{min} \sum_{i,j=1}^{n} M{S}_{ij}^{m}{\Vert {q}_{m}^{i}-{q}_{m}^{j}\Vert }_{2}+{\text{Tr}}{\left({Q}_{m}-MD\right)}^{T}{U}_{m}\left({Q}_{m}-MD\right)\end{array}$$

For the disease space, the objective function was defined as Eq. (9). Here $Qd$ is a label matrix to be solved.

9 $$\begin{array}{c}\underset{{Q}_{d}}{min} \sum_{i,j=1}^{m} D{S}_{ij}^{d}{\Vert {q}_{d}^{i}-{q}_{d}^{j}\Vert }_{2}+{\text{Tr}}{\left({Q}_{d}-M{D}^{T}\right)}^{T}{U}_{d}\left({Q}_{d}-M{D}^{T}\right)\end{array}$$

These two parts of the objective function were solved by an iterative algorithm until convergence, which was described in details in in the original article by Liang et al. [42] and will not be repeated here. We also compared the matrix factorization with semi-supervised learning method to demonstrate their impact on model prediction powers (Additional file 1: Table S2). Briefly, even with the same miRNA similarity network, semi-supervised learning (adopted by DNI-MDCAP) showed better performance than matrix factorization (adopted by LE-MDCAP) in distinguishing between causal and non-causal miRNA-disease associations. Besides, semi-supervised learning seems more compatible to the network imputation, as a noticeable gap of performance can be observed for DNI-MDCAP with and without network imputation, while such gap is much smaller for LE-MDCAP.

### The combined DNI-MDCAP prediction score

For each miRNA similarity matrix input $MS$, the corresponding ${Q}_{m}$ and ${Q}_{d}$ obtained above were finally integrated to generate a predictive correlation score matrix ${MD\prime}$, as specified in Eq. (10).

10 $$\begin{array}{c}M{D}^{\mathrm{^{\prime}}}=\frac{\left({Q}_{m}+{Q}_{d}^{T}\right)}{2}\end{array}$$

Notably, in this process, only one miRNA similarity matrix will be considered at a time. Since six miRNA similarity matrices ${MS}_{S}$, ${MS}_{E}$, $GM$, ${MS}_{P}$, ${MS}_{gene}$, ${MS}_{tf}$ were considered, this process was conducted six times with different input $MS$ to obtain six prediction score matrices, that is, ${MD{\prime}}_{S}$, ${MD{\prime}}_{E}$, ${MD{\prime}}_{GM}$, ${MD{\prime}}_{P}$, ${MD{\prime}}_{gene}$, ${MD{\prime}}_{tf}$. Finally, the combined predictive score matrix ${MD{\prime}}_{combined}$ was obtained based on the weighted sum of the above six predictive scores. The wights were optimized with a step size of 0.05. The final combined predictive score matrix was calculated as ${MD{\prime}}_{combined}$ = 0.15 ${MD{\prime}}_{S}$ + 0.1 ${MD{\prime}}_{E}$ + 0.05 ${MD{\prime}}_{GM}$ + 0.25 ${MD{\prime}}_{P}$ + 0.2 ${MD{\prime}}_{gene}$ + 0.25 ${MD{\prime}}_{tf}$.

### Model evaluation and online server construction

To assess the predictive accuracy of DNI-MDCAP, we conducted both independent test and ten-fold cross-validation. It is important to clarify that the independent test dataset was not used in feature selection and score integration weight optimization processes to avoid overfitting. Additionally, we compared DNI-MDCAP with previously established prediction models, MDCAP and LE-MDCAP, in discriminating causal and non-causal miRNA-disease associations. DNI-MDCAP, MDCAP and LE-MDCAP were trained and tested on the same training and test samples. We also compared DNI-MDCAP with recent models designed for general (not for causal) miRNA-disease association. In this performance comparison, all models were re-trained and tested on the filtered training and testing sets of DNI-MDCAP. More specifically, compared to the original training and testing sets, in order to ensure to a fair comparison, we removed all miRNAs and diseases that have not been considered by the previous models (among both positive and negative samples), and fixed the positive-to-negative ratio to 1:5 in the causal-versus-non-disease test.

We plotted the receiver operating characteristic (ROC) curve by calculating the true positive rate (TPR) and false positive rate (FPR) across various thresholds and measured the performance via area under the ROC curve (AUROC).

For the users’ convenience, DNI-MDCAP is accessible as an online server that was implemented by HTML + PHP + Apache framework.

## Results

### Overall performance evaluation of DNI-MDCAP

**Fig. 2:**
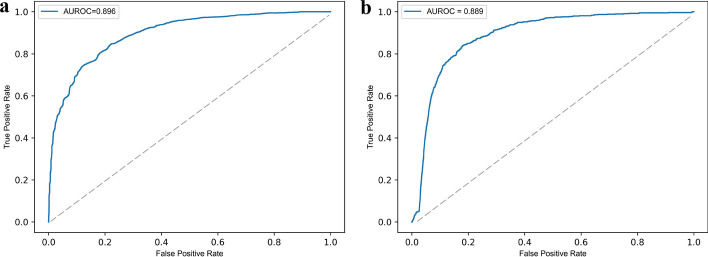
ROC curves performed by DNI-MDCAP: **a** ROC curve of tenfold cross-validation. **b** ROC curve of independent test

We first assessed the ability of DNI-MDCAP to distinguish causal miRNA-disease associations (causal) from miRNA-disease pairs with no association (non-disease) using both ten-fold cross-validation and independent test. The evaluation was based on known causal miRNA-disease associations in the HMDD v3.2 database. We randomly selected approximately one-fifth of the known miRNA-disease causal associations as the independent test set (before network imputation), while the remaining four-fifths were used as the training set. Similarly, in each round of ten-fold cross-validation, the known miRNA-disease causal associations were divided into consistent proportions for the training and the test sets. To prevent data leakage, the model’s parameter selection was only based on the training samples. Then, AUROC was introduced as the measure of the predictive model’s performance. DNI-MDCAP achieved an AUROC value of 0.896 in the ten-fold cross-validation (Fig. 2a) and an AUROC value of 0.889 in the independent test (Fig. 2b). These results have demonstrated the competitive performance of our method in predicting potential causal miRNA-disease associations.

### DNI-MDCAP accurately distinguishes causal and non-causal miRNA-disease associations

**Fig. 3:**
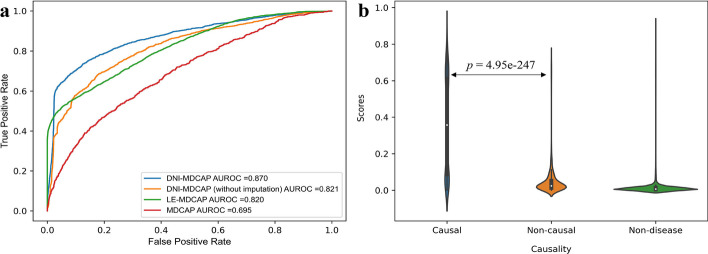
Improved predictive performance of DNI-MDCAP: **a** ROC curves of DNI-MDCAP (with or without imputation) and previous models in discriminating causal miRNA-disease associations from the non-causal associations. **b** Violin plots of DNI-MDCAP showing the distribution of model prediction scores in the causal, non-causal and non-disease groups

Discriminating causal miRNA-disease associations from non-causal ones is more challenging compared to distinguishing causal associations from unrelated miRNA-disease pairs. To validate the DNI-MDCAP model, we categorized all miRNA-disease pairs in the dataset into three groups: causal miRNA-disease associations (causal), non-causal miRNA-disease associations (non-causal), and unrelated miRNA-disease pairs (non-disease). To test the DNI-MDCAP’s ability to discern causal associations from non-causal ones, in this sub-section, we considered ‘causal’ as positive samples and ‘non-causal’ as negative samples for method evaluation. To ensure the fairness of the performance comparison, the above DNI-MDCAP prediction scores were directly applied to this dataset, and no re-training or re-optimization of the model was conducted during this evaluation. The results indicated that the previously published MDCAP model exhibited limited discriminative power (AUROC = 0.695), whereas our previous predictive model LE-MDCAP showed significant improvement (AUROC = 0.820). Notably, DNI-MDCAP outperformed these two existing models in distinguishing between causal and non-causal miRNA-disease associations, achieving an AUROC of 0.870. To further verify the usefulness of deep network imputation, we also performed an ablation test using the raw miRNA similarity network and miRNA-disease network instead of the computationally imputed ones. The results suggested that the AUROC of DNI-MDCAP decreased to 0.821 without network imputation (Fig. 3a). Besides, we assessed the statistical significance of the variation in prediction scores between the three miRNA-disease groups by performing the Wilcoxon rank-sum test. The analysis revealed a statistically highly significant distinction (*p* = 4.95e-247) between the predicted scores of the causal and non-causal associations (Fig. 3b). In summary, the above results collectively manifested that DNI-MDCAP was highly effective in distinguishing causal miRNA-disease associations from non-causal associations, exhibiting excellent predictive performance. Because our previous benchmarking was performed in 2019 [24], we also compared the efficacy of DNI-MDCAP with those of recently published miRNA-disease prediction models, including SMALF [21], GATMDA [22], and CFESMDA [23]. Several previous models show better AUROC in the casual-versus-non-disease test, but they only show weak to moderate prediction performance in the casual-versus-non-causal test (Additional file 1: Fig. S1a, b). We also used violin plots to depict the distribution of the prediction scores of different models between the causal, non-causal and non-disease groups (Additional file 1: Fig. S1c–f). The results suggest that DNI-MDCAP recognizes causal miRNA-disease associations with significantly higher prediction scores than non-causal associations, while the differences in prediction score between causal and non-causal associations are not so obvious for other previous models. Together, the performance comparison results suggest that, as an ad hoc model for causal miRNA-disease association prediction, DNI-MDCAP indeed showed superior performances in distinguishing causal miRNA-disease associations from non-causal and non-disease associations.

### Case study of DNI-MDCAP prediction results with latest literature

We conducted case studies by examining the top predictions of DNI-MDCAP and validating these predictions with recent literature records that were not included in the HMDD v3.2 dataset. These new literature records were not included in the training or test dataset of DNI-MDCAP to ensure their independence. Therefore, they provided an additional chance to evaluate DNI-MDCAP’s performance, complementing the conventional AUROC evaluation. To start with, we investigated whether the predictions from DNI-MDCAP are helpful in identifying new causal miRNAs for a specific disease. Diabetic nephropathy is a common complication in diabetic patients, often leading to end-stage renal failure and posing severe health risks [43]. Understanding the molecular mechanisms underlying diabetic nephropathy is crucial for the development of effective therapeutic interventions to alleviate its symptoms. In this study, we utilized the prediction scores generated by DNI-MDCAP to identify causal miRNAs that may potentially involve in the pathogenic pathways of diabetic nephropathy. Table 1 indicates that all of the top five causal miRNA-diabetic nephropathies associations predicted by DNI-MDCAP were supported by recent literature evidence. Furthermore, seven out of the top ten prediction results were supported by the literature. Among all potentially causal miRNAs, hsa-mir-30c had the highest score of 0.3931. Notably, miR-30c-5p was reported to effectively inhibit epithelial-mesenchymal transition and kidney fibrosis, playing a pivotal role in preventing the progression of diabetic nephropathy [44]. Another study suggested that miR-155 promoted hyperglycemia-induced podocyte inflammation by targeting SIRT1, leading to impaired renal function and exacerbated renal pathological changes [45]. Similarly, miR-29a has been suggested to contribute to the pathogenesis of tubulointerstitial fibrosis by modulating the CB1R pathway, thereby accelerating the progression of diabetic nephropathy [46]. A publication in 2020 demonstrated that the ablation of miR-214 from renal proximal tubules prevented a decrease in ULK1 expression and autophagy impairment in diabetic kidneys, resulting in less renal hypertrophy and albuminuria [8]. Furthermore, miR-20a was indicated to target CXCL6 and modulate JAK/STAT3 signaling, exerting an inhibitory effect on diabetic nephropathy [47]. All these articles confirmed that the top causal miRNA-diabetic nephropathies associations predicted by DNI-MDCAP were practical and instructive.

**Table 1:** Top 10 causal miRNA-diabetic nephropathies associations predictions by DNI-MDCAP and their literature verification

Ranking	MiRNA	Score	PMID	Published year
1	hsa-mir-30c	0.3931	32096183	2020
2	hsa-mir-155	0.0570	33748285	2021
3	hsa-mir-29a	0.0560	30642005	2020
4	hsa-mir-214	0.0481	32804155	2020
5	hsa-mir-20a	0.0461	32868134	2021
6	hsa-mir-34a	0.0405	36345369	2022
7	hsa-mir-210	0.0404	NA	NA
8	hsa-mir-17	0.0393	34014023	2021
9	hsa-mir-15a	0.0392	NA	NA
10	hsa-mir-182	0.0368	NA	NA

To ensure the independence, causal associations that have already been included in the HMDD v3.2 are not listed here

In addition, we conducted another case study to evaluate if DNI-MDCAP could help identify potential causal associations involving a specific miRNA of interest. Previous studies have implicated hsa-mir-193a in several disease processes, including the progression of non-alcoholic fatty liver disease [48] and tumor cell metabolism [49] among others. Recognizing its relevance, we delved further into more causal relationships between hsa-mir-193a and diseases, as revealed by the top predictions from DNI-MDCAP (Table 2). Out of the top five predictions, four have been validated by recent literature evidence. Moreover, among the top ten prediction results, eight have been validation by the recent literature. The association with hepatocellular carcinoma (HCC) achieved the highest score of 0.6887 among all potential diseases. In HCC cells, miR-193a was regulated by Mig-6, leading to autophagy inhibition, which presents a potential therapeutic target for HCC treatment [50]. In colorectal cancer, miR-193a functioned through the miR-193a-5p/PIK3R3/AKT axis, playing a role in the initiation and progression of the disease [51]. Furthermore, a study in 2020 unveiled the involvement of miR-193a in a competitive endogenous RNA regulatory pathway in breast cancer, suggesting a novel strategy for breast cancer treatment [52]. In bladder cancer cells, miR-193a was identified as an upstream target of ZNFX1-AS1, promoting tumor cell proliferation, migration, and invasion [53]. These articles collectively verified the involvement of hsa-mir-193a in the onset and progression of diseases predicted by DNI-MDCAP, and supported the causality of the predicted associations.

**Table 2:** Top ten causal hsa-mir-193a-diseases predictions by DNI-MDCAP and their literature verification

Ranking	Disease	Score	PMID	Published year
1	Carcinoma, hepatocellular	0.6887	35165519	2022
2	Colorectal neoplasms	0.6609	33317596	2020
3	Breast neoplasms	0.6256	32497022	2020
4	Urinary bladder neoplasms	0.5584	32432735	2020
5	Neuralgia	0.3704	NA	NA
6	Glioma	0.1201	33968717	2021
7	Carcinoma, non-small-cell lung	0.1169	36183046	2022
8	Uterine cervical neoplasms	0.0921	NA	NA
9	Glioblastoma	0.0661	30304561	2019
10	Pancreatic neoplasms	0.0610	36476048	2023

To ensure the independence, causal associations that have already been included in the HMDD v3.2 are not listed here

### DNI-MDCAP server

**Fig. 4:**
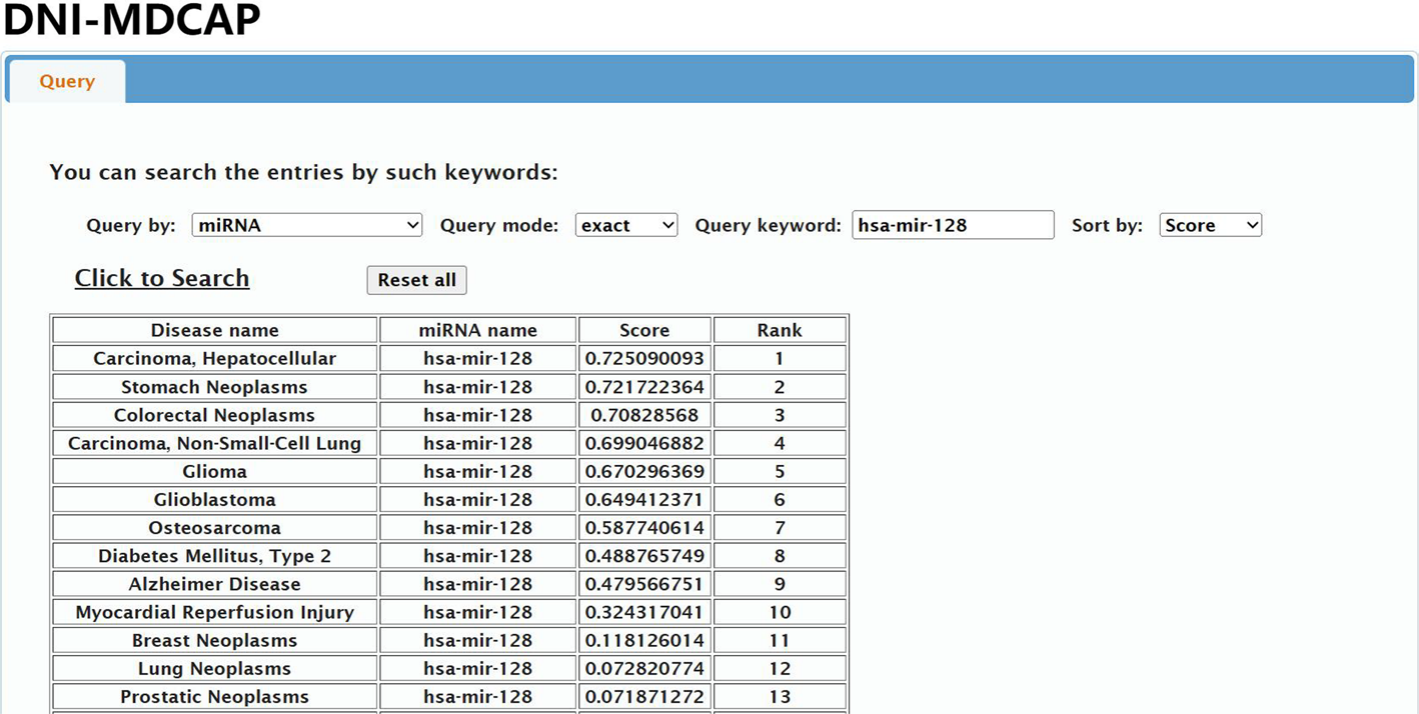
The query interface and sample result of DNI-MDCAP server

We have developed a user-friendly web server interface (Fig. 4) for DNI-MDCAP (available at http://www.rnanut.net/DNIMDCAP/) to facilitate easy querying. Users can access prediction results by entering miRNA names or disease keywords, supporting both precise and fuzzy search modes. Furthermore, users have the flexibility to choose the ranking criteria for the prediction results, including miRNA rank (miRNA), disease rank (Disease), or score rank (Score, default), enabling convenient identification and prioritization of the most likely causal miRNAs associated with specific diseases.

## Discussion

In recent years, the field of bioinformatics has witnessed a surge in studies exploring the connections between miRNAs and diseases. Many of the identified miRNAs are found to undergo changes in expression or localization as the body transitions from a physiological to a pathological state [54, 55]. These miRNAs are often observed in a passive role during disease progression, and their association with diseases are referred as non-causal miRNA-disease associations. Despite not being directly involved in the onset and progression of diseases, these miRNAs are widely used as biomarkers in clinical settings for purposes such as increasing diagnostic sensitivity, evaluating treatment response, and predicting prognosis [56]. However, non-causal miRNA-disease associations do not offer provide insight into the underlying mechanisms of the disease, nor do they serve as viable therapeutic targets. It is crucial to identify causal miRNAs that directly contribute to the development of diseases and are actively involved in pathogenesis. To address the need for the identification of disease causal miRNAs, we here proposed the prediction method DNI-MDCAP, which enriched existing models for predicting the causal miRNA-disease associations. Notably, DNI-MDCAP exhibited significant advantages in distinguishing causal miRNA-disease from non-causal ones, outperforming MDCAP and our previously proposed LE-MDCAP. The results of the case studies provided supplemental confirmation of the predictive reliability of DNI-MDCAP. Ablation experiment also suggested both the extended miRNA similarity metrics, network imputation and semi-supervised learning substantially contribute to the performance of DNI-MDCAP. Another advantage of DNI-MDCAP is that it performs causal-versus-non-disease and causal-versus-non-causal discrimination using the same model, avoid complicated model retraining and design. In summary, DNI-MDCAP has displayed dependable predictive performance in distinguishing causal and non-causal miRNA disease associations, enabling optimal selection of causal miRNA-disease associations.

Nonetheless, there is still room for improvement in DNI-MDCAP. One practical limitation of DNI-MDCAP is its limited ability to predict diseases, as it relies on known causal miRNA-disease association datasets. As a result, it may not be applicable to novel diseases for which there are few even no known causally associated miRNAs. Nevertheless, as more disease-causal miRNA annotation data become available, it can be anticipated that the predictive performance and disease coverage of DNI-MDCAP will be improved in future studies. Secondly, in the ten-fold cross-validation and independent test, MDCAP predicted an AUROC of 0.928 and 0.925 for miRNA-disease association, respectively, which was superior to DNI-MDCAP (AUROC of 0.896 and 0.889, respectively). As a consequence, the algorithm model needs further upgrade in the future to better balance the predictive performance of causal-versus-non-disease and causal-versus-non-causal discrimination. An alternative solution should be applying distinct computational frameworks for these two tasks to further improve the performances in the future, in order to optimize the performance of each task, respectively. Integrating miRNA and disease feature dimensions through deep learning may also contribute to more accurate similarity measurements, enhancing the model’s predictive performance. Finally, causal miRNAs show bidirectional impacts on diseases, i.e., either promoting or inhibiting disease progression. Therefore, we believe that in future predictions of miRNA-disease causality, it would be beneficial to consider the prediction of causal association categories (i.e., disease promoting or inhibiting). This addition would help identify the precise direction for intervening in disease treatment targets, leading to more accurate and effective therapeutic interventions.

## Conclusions

DNI-MDCAP is a computational model based on diverse miRNA similarity metrics, deep graph embedding assisted-network imputation and semi-supervised learning algorithm, designed to predict potential causal miRNA-disease associations. It exhibited reliable predictive performance, effectively distinguishing between causal and non-causal miRNA-disease associations as well as causal and unrelated ones. The accuracy of its predictions has been validated by recently published literature. This advance is helpful to refine our undemanding of causality in miRNA-disease associations and provides meaningful clues for potential causal associations between miRNAs and diseases. Consequently, it provides useful guidance for exploring novel targets for disease treatment.

### Availability and requirements

- Project name: DNI-MDCAP
- Project home page: http://www.rnanut.net/DNIMDCAP/
- Operating system: Platform independent
- Programming language: Python, R
- Other requirements: None
- License: GNU GPL
- Any restrictions to use by non-academics: None.

### Supplementary Information

**Additional file 1: Fig. S1.** Comparison of predictive performance between DNI-MDCAP and other previous models: All models were re-trained and tested on the filtered training and testing sets of DNI-MDCAP. More specifically, compared to the original training and testing sets, in order to ensure to a fair comparison, we removed all miRNAs and diseases that have not been considered by the previous models (among both positive and negative samples), and fixed the positive-to-negative ratio to 1:5 in the causal-versus-non-disease test. It is also noteworthy that the previous models were designed for general miRNA-disease association prediction without a specification of causality. **a** ROC curves of DNI-MDCAP and the previous models in discriminating causal miRNA-disease associations from the non-causal associations. **b** ROC curves of DNI-MDCAP and the previous models in discriminating causal miRNA-disease associations from the non-disease associations. **c**–**f** Violin plots showing the distribution of prediction scores of different models, between the causal, non-causal and non-disease groups. **Table S1.** Performance comparison using different miRNA similarity metrics. **Table S2.** Ablation experiments comparing the components in the computational frameworks of LE-MDCAP and DNI-MDCAP.

## Data Availability

DNI-MDCAP web server is freely available at http://www.rnanut.net/DNIMDCAP/. The data and code used in the current study are available at: https://github.com/zhouqoiii/DNI-MDCAP.

## Abbreviations

miRNA: MicroRNA
HMDD: Human MiRNA Disease Database
MDCAP: MiRNA-Disease Causal Association Predictor
LE-MDCAP: Levenshtein-distance Enhanced MiRNA-Disease Causal Association Predictor
DNI-MDCAP: Deep Network Imputation-assisted MiRNA-Disease Causal Association Predictor
TF: Transcription factor
DAG: Directed acyclic graph
ROC: Receiver operating characteristic
TPR: True positive rate
FPR: False positive rate
AUROC: Area under the receiver operating characteristic curve

## References

[1] Bartel DP (2009). MicroRNAs: target recognition and regulatory functions. Cell.

[2] Lu TX, Rothenberg ME (2018). MicroRNA. J Allergy Clin Immunol.

[3] Peng Y, Chen FF, Ge J, Zhu JY, Shi XE, Li X, Yu TY, Chu GY, Yang GS (2016). miR-429 inhibits differentiation and promotes proliferation in porcine preadipocytes. Int J Mol Sci.

[4] Fan L, Lai R, Ma N, Dong Y, Li Y, Wu Q, Qiao J, Lu H, Gong L, Tao Z (2021). miR-552-3p modulates transcriptional activities of FXR and LXR to ameliorate hepatic glycolipid metabolism disorder. J Hepatol.

[5] Guo FH, Guan YN, Guo JJ, Zhang LJ, Qiu JJ, Ji Y, Chen AF, Jing Q (2022). Single-Cell transcriptome analysis reveals embryonic endothelial heterogeneity at spatiotemporal level and multifunctions of microRNA-126 in mice. Arterioscler Thromb Vasc Biol.

[6] Zhao D, Wu K, Sharma S, Xing F, Wu SY, Tyagi A, Deshpande R, Singh R, Wabitsch M, Mo YY (2022). Exosomal miR-1304-3p promotes breast cancer progression in African Americans by activating cancer-associated adipocytes. Nat Commun.

[7] Zhang Y, Cai Y, Zhang H, Zhang J, Zeng Y, Fan C, Zou S, Wu C, Fang S, Li P (2021). Brown adipose tissue transplantation ameliorates diabetic nephropathy through the miR-30b pathway by targeting Runx1. Metabolism.

[8] Ma Z, Li L, Livingston MJ, Zhang D, Mi Q, Zhang M, Ding HF, Huo Y, Mei C, Dong Z (2020). p53/microRNA-214/ULK1 axis impairs renal tubular autophagy in diabetic kidney disease. J Clin Invest.

[9] Zhang X, Ji R, Liao X, Castillero E, Kennel PJ, Brunjes DL, Franz M, Mobius-Winkler S, Drosatos K, George I (2018). MicroRNA-195 regulates metabolism in failing myocardium via alterations in Sirtuin 3 expression and mitochondrial protein acetylation. Circulation.

[10] Kandettu A, Radhakrishnan R, Chakrabarty S, Sriharikrishnaa S, Kabekkodu SP (2020). The emerging role of miRNA clusters in breast cancer progression. Biochim Biophys Acta Rev Cancer.

[11] Zhao W, Zhang R, Zang CY, Zhang LF, Zhao R, Li QC, Yang ZJ, Feng Z, Zhang W, Cui RT (2022). Exosome derived from mesenchymal stem cells alleviates pathological scars by inhibiting the proliferation, migration and protein expression of fibroblasts via delivering miR-138-5p to target SIRT1. Int J Nanomed.

[12] Uppala SN, Tryphena KP, Naren P, Srivastava S, Singh SB, Khatri DK (2023). Involvement of miRNA on epigenetics landscape of Parkinson's disease: from pathogenesis to therapeutics. Mech Ageing Dev.

[13] Arroyo AB, Aguila S, Fernandez-Perez MP, Reyes-Garcia AML, Reguilon-Gallego L, Zapata-Martinez L, Vicente V, Martinez C, Gonzalez-Conejero R (2021). miR-146a in cardiovascular diseases and sepsis: an additional burden in the inflammatory balance?. Thromb Haemost.

[14] Zhang MW, Shen YJ, Shi J, Yu JG (2020). MiR-223-3p in cardiovascular diseases: a biomarker and potential therapeutic target. Front Cardiovasc Med.

[15] Pogue AI, Lukiw WJ (2021). microRNA-146a-5p, neurotropic viral infection and prion disease (PrD). Int J Mol Sci.

[16] Mori MA, Ludwig RG, Garcia-Martin R, Brandao BB, Kahn CR (2019). Extracellular miRNAs: from biomarkers to mediators of physiology and disease. Cell Metab.

[17] Fu L, Peng Q (2017). A deep ensemble model to predict miRNA-disease association. Sci Rep.

[18] Chen X, Zhu CC, Yin J (2019). Ensemble of decision tree reveals potential miRNA-disease associations. PLoS Comput Biol.

[19] Chen X, Liu MX, Yan GY (2012). RWRMDA: predicting novel human microRNA-disease associations. Mol Biosyst.

[20] Chen H, Zhang Z (2013). Similarity-based methods for potential human microRNA-disease association prediction. BMC Med Genom.

[21] Liu D, Huang Y, Nie W, Zhang J, Deng L (2021). SMALF: miRNA-disease associations prediction based on stacked autoencoder and XGBoost. BMC Bioinf.

[22] Li G, Fang T, Zhang Y, Liang C, Xiao Q, Luo J (2022). Predicting miRNA-disease associations based on graph attention network with multi-source information. BMC Bioinf.

[23] Hu X, Yin Z, Zeng Z, Peng Y (2023). Prediction of miRNA-disease associations by cascade forest model based on stacked autoencoder. Molecules.

[24] Huang Z, Liu L, Gao Y, Shi J, Cui Q, Li J, Zhou Y (2019). Benchmark of computational methods for predicting microRNA-disease associations. Genome Biol.

[25] Xue H, Shen X, Pan W (2023). Causal inference in transcriptome-wide association studies with invalid instruments and GWAS summary data. J Am Stat Assoc.

[26] Belyaeva A, Squires C, Uhler C (2021). DCI: learning causal differences between gene regulatory networks. Bioinformatics.

[27] Huang Z, Shi J, Gao Y, Cui C, Zhang S, Li J, Zhou Y, Cui Q (2019). HMDD v3.0: a database for experimentally supported human microRNA-disease associations. Nucleic Acids Res.

[28] Gao Y, Jia K, Shi J, Zhou Y, Cui Q (2019). A computational model to predict the causal miRNAs for diseases. Front Genet.

[29] Huang Z, Han Y, Liu L, Cui Q, Zhou Y (2021). LE-MDCAP: a computational model to prioritize causal miRNA-disease associations. Int J Mol Sci.

[30] Wang KR, McGeachie MJ (2022). DisiMiR: predicting pathogenic miRNAs using network influence and miRNA conservation. Noncod RNA.

[31] Medical Subject Headings database [https://www.nlm.nih.gov/mesh]

[32] Kozomara A, Birgaoanu M, Griffiths-Jones S (2019). miRBase: from microRNA sequences to function. Nucleic Acids Res.

[33] Tong Z, Cui Q, Wang J, Zhou Y (2019). TransmiR v20: an updated transcription factor-microRNA regulation database. Nucleic Acids Res.

[34] Huang HY, Lin YC, Li J, Huang KY, Shrestha S, Hong HC, Tang Y, Chen YG, Jin CN, Yu Y (2020). miRTarBase 2020: updates to the experimentally validated microRNA-target interaction database. Nucleic Acids Res.

[35] Lorenzi L, Chiu HS, Cobos FA, Gross S, Volders PJ, Cannoodt R, Nuytens J, Vanderheyden K, Anckaert J, Lefever S (2021). The RNA Atlas expands the catalog of human non-coding RNAs. Nat Biotechnol.

[36] Kehl T, Kern F, Backes C, Fehlmann T, Stockel D, Meese E, Lenhof HP, Keller A (2020). miRPathDB 2.0: a novel release of the miRNA Pathway Dictionary Database. Nucleic Acids Res.

[37] Lu M, Zhang Q, Deng M, Miao J, Guo Y, Gao W, Cui Q (2008). An analysis of human microRNA and disease associations. PLoS ONE.

[38] van Laarhoven T, Nabuurs SB, Marchiori E (2011). Gaussian interaction profile kernels for predicting drug-target interaction. Bioinformatics.

[39] Wang D, Wang J, Lu M, Song F, Cui Q (2010). Inferring the human microRNA functional similarity and functional network based on microRNA-associated diseases. Bioinformatics.

[40] Wu XB, Zhou Y (2022). GE-Impute: graph embedding-based imputation for single-cell RNA-seq data. Brief Bioinform.

[41] Mikolov T SI, Chen K, Corrado G, Dean J.: Distributed representations of words and phrases and their compositionality. In: Proceedings of the 26th international conference on neural information processing systems. Lake Tahoe, Nevada: Curran Associates Inc; 2013. p. 3111–9.

[42] Liang C, Yu S, Wong KC, Luo J (2018). A novel semi-supervised model for miRNA-disease association prediction based on [Formula: see text]-norm graph. J Transl Med.

[43] Thomas MC, Brownlee M, Susztak K, Sharma K, Jandeleit-Dahm KA, Zoungas S, Rossing P, Groop PH, Cooper ME (2015). Diabetic kidney disease. Nat Rev Dis Primers.

[44] Gao BH, Wu H, Wang X, Ji LL, Chen C (2020). MiR-30c-5p inhibits high glucose-induced EMT and renal fibrogenesis by down-regulation of JAK1 in diabetic nephropathy. Eur Rev Med Pharmacol Sci.

[45] Wang X, Gao Y, Yi W, Qiao Y, Hu H, Wang Y, Hu Y, Wu S, Sun H, Zhang T (2021). Inhibition of miRNA-155 alleviates high glucose-induced podocyte inflammation by targeting SIRT1 in diabetic mice. J Diabetes Res.

[46] Tung CW, Ho C, Hsu YC, Huang SC, Shih YH, Lin CL (2019). MicroRNA-29a attenuates diabetic glomerular injury through modulating cannabinoid receptor 1 signaling. Molecules.

[47] Wang SZ, Zhang YL, Shi HB (2021). Potential repressive impact of microRNA-20a on renal tubular damage in diabetic kidney disease by targeting C-X-C motif chemokine ligand 6. Arch Med Res.

[48] Johnson K, Leary PJ, Govaere O, Barter MJ, Charlton SH, Cockell SJ, Tiniakos D, Zatorska M, Bedossa P, Brosnan MJ (2022). Increased serum miR-193a-5p during non-alcoholic fatty liver disease progression: diagnostic and mechanistic relevance. JHEP Rep.

[49] Asl ER, Amini M, Najafi S, Mansoori B, Mokhtarzadeh A, Mohammadi A, Lotfinejad P, Bagheri M, Shirjang S, Lotfi Z (2021). Interplay between MAPK/ERK signaling pathway and MicroRNAs: a crucial mechanism regulating cancer cell metabolism and tumor progression. Life Sci.

[50] Qu L, Tian Y, Hong D, Wang F, Li Z (2022). Mig-6 inhibits autophagy in HCC cell lines by modulating miR-193a-3p. Int J Med Sci.

[51] Xu H, Liu Y, Cheng P, Wang C, Liu Y, Zhou W, Xu Y, Ji G (2020). CircRNA_0000392 promotes colorectal cancer progression through the miR-193a-5p/PIK3R3/AKT axis. J Exp Clin Cancer Res.

[52] Li J, Zeng T, Li W, Wu H, Sun C, Yang F, Yang M, Fu Z, Yin Y (2020). Long non-coding RNA SNHG1 activates HOXA1 expression via sponging miR-193a-5p in breast cancer progression. Aging (Albany NY).

[53] Wu JP, Zhang GY, Sun XZ (2020). LncRNA ZNFX1-AS1 targeting miR-193a-3p/SDC1 regulates cell proliferation, migration and invasion of bladder cancer cells. Eur Rev Med Pharmacol Sci.

[54] Kai K, Dittmar RL, Sen S (2018). Secretory microRNAs as biomarkers of cancer. Semin Cell Dev Biol.

[55] Wiedrick JT, Phillips JI, Lusardi TA, McFarland TJ, Lind B, Sandau US, Harrington CA, Lapidus JA, Galasko DR, Quinn JF (2019). Validation of MicroRNA biomarkers for Alzheimer's Disease in human cerebrospinal fluid. J Alzheimers Dis.

[56] Zhang HD, Jiang LH, Sun DW, Hou JC, Ji ZL (2018). CircRNA: a novel type of biomarker for cancer. Breast Cancer Tokyo.

