# Supplementary material for: DNI-MDCAP: improvement of causal MiRNA-disease association prediction based on deep network imputation
Source: BMC Bioinformatics. 2024 Jan 12;25:22. doi: 10.1186/s12859-024-05644-6 (PMC10785389; doi:10.1186/s12859-024-05644-6)
Supplement: Supplementary file 1 — Additional file 1: Fig. S1. Comparison of predictive performance between DNI-MDCAP and other previous models: All models were re-trained and tested on the filtered training and testing sets of DNI-MDCAP. More specifically, compared to the original training and testing sets, in order to ensure to a fair comparison, we removed all miRNAs and diseases that have not been considered by the previous models (among both positive and negative samples), and fixed the positive-to-negative ratio to 1:5 in the causal-versus-non-disease test. It is also noteworthy that the previous models were designed for general miRNA-disease association prediction without a specification of causality. a ROC curves of DNI-MDCAP and the previous models in discriminating causal miRNA-disease associations from the non-causal associations. b ROC curves of DNI-MDCAP and the previous models in discriminating causal miRNA-disease associations from the non-disease associations. c–f Violin plots showing the distribution of prediction scores of different models, between the causal, non-causal and non-disease groups. Table S1. Performance comparison using different miRNA similarity metrics. Table S2. Ablation experiments comparing the components in the computational frameworks of LE-MDCAP and DNI-MDCAP. [file 12859_2024_5644_MOESM1_ESM.pdf]

Additional file 1

DNI-MDCAP: Improvement of Causal MiRNA-Disease Association Prediction Based on Deep Network Imputation

Supplementary Figure

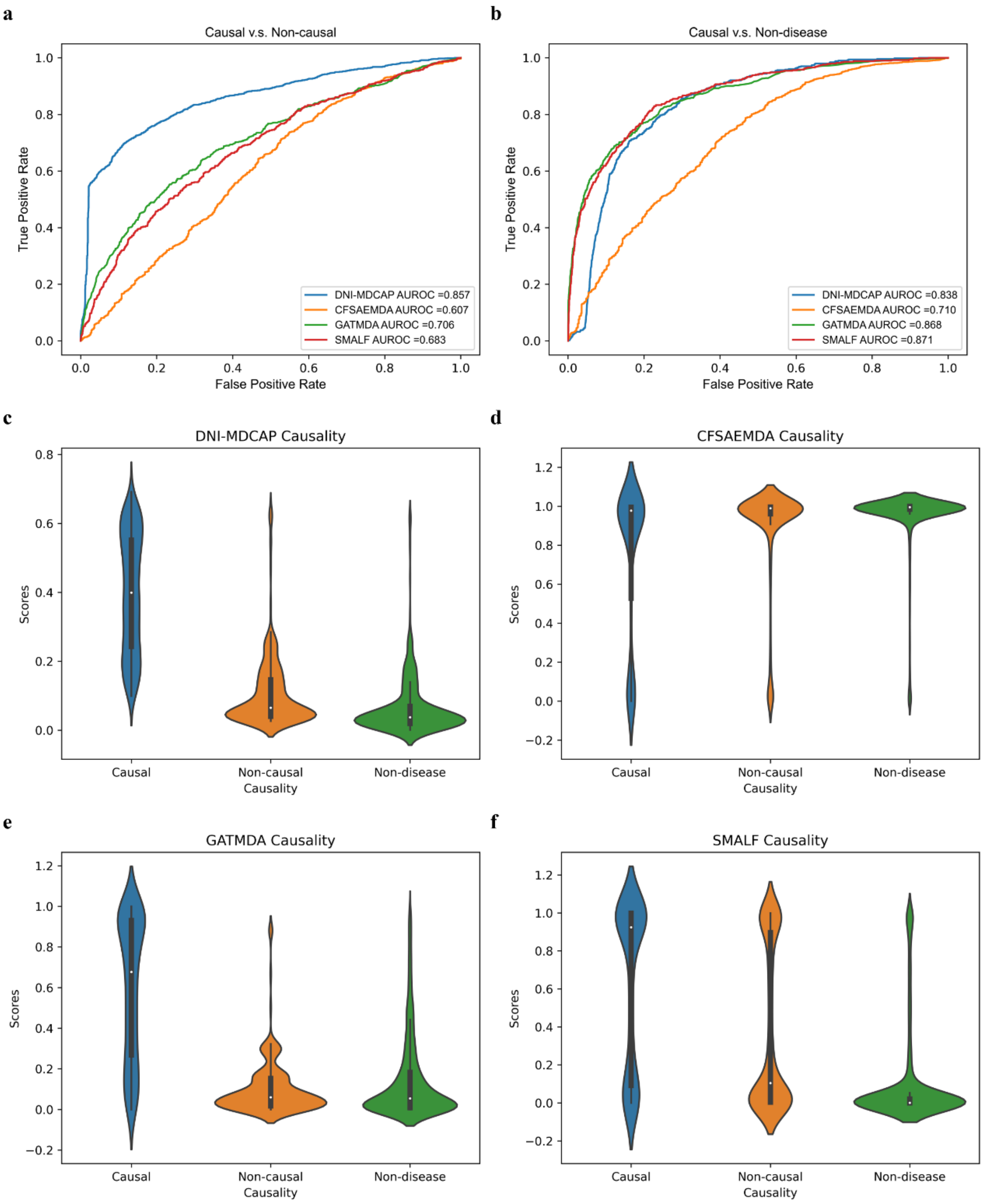

Figure S1. Comparison of predictive performance between DNI-MDCAP and other previous models: All models were re-trained and tested on the filtered training

and testing sets of DNI-MDCAP. More specifically, compared to the original training and testing sets, in order to ensure to a fair comparison, we removed all miRNAs and diseases that have not been considered by the previous models (among both positive and negative samples), and fixed the positive-to-negative ratio to 1:5 in the causal-versus-non-disease test. It is also noteworthy that the previous models were designed for general miRNA-disease association prediction without a specification of causality. (a) ROC curves of DNI-MDCAP and the previous models in discriminating causal miRNA-disease associations from the non-causal associations. (b) ROC curves of DNI-MDCAP and the previous models in discriminating causal miRNA-disease associations from the non-disease associations. (c)-(f) Violin plots showing the distribution of prediction scores of different models, between the causal, non-causal and non-disease groups.

Supplementary Tables

Table S1. Performance comparison using different miRNA similarity metrics.

MS	Replacement	AUROC (causal vs. non-causal)	AUROC (causal vs. non-disease)
**Default#**		0.8674	0.892
	Cosine	0.8635	0.891
	Euclidean	0.8625	0.893
MS_E	Spearman	0.8631	0.891
	Pearson	0.8631	0.891
	Tanimoto*	0.8650	0.887
	Cosine	0.8679	0.893
	Euclidean	0.8680	0.893
MS_P	Spearman	0.8680	0.893
	Pearson	0.8680	0.893
	Tanimoto*	0.8689	0.891
	Cosine	0.8665	0.893
MS_{tf}	Euclidean	0.8664	0.893
	Spearman	0.8665	0.893
	Pearson	0.8664	0.893
	Tanimoto	0.8665	0.893
	Cosine	0.8569	0.891
MS_{gene}	Euclidean	0.8570	0.891
	Spearman	0.8569	0.891
	Pearson	0.8570	0.891
	Tanimoto	0.8572	0.891

#: similarity metrics based on Levenshitein distance except GM.

*: chosen in this study.

Table S2. Ablation experiments comparing the components in the computational frameworks of LE-MDCAP and DNI-MDCAP.

Model	AUROC (causal vs. non-causal)	AUROC (causal vs. non-disease)
LE-MDCAP	0.820	0.906
LE-MDCAP with imputation	0.821	0.885
LE-MDCAP with DNI-MDCAP's imputed miRNA network	0.827	0.874
DNI-MDCAP without imputation	0.821	0.891
DNI-MDCAP	0.870	0.889
